# Supplementary material for: Learning curve and surgical outcome of robotic assisted colorectal surgery with ERAS program
Source: Sci Rep. 2022 Nov 29;12:20566. doi: 10.1038/s41598-022-24665-w (PMC9709162; doi:10.1038/s41598-022-24665-w)
Supplement: Supplementary file 1 — Supplementary Information 1. [file 41598_2022_24665_MOESM1_ESM.zip › LSC Raw data-Ñ[▒K/076/N_ñw╝╨░O▒Kñσ.pdf]

W52 / W52

臺中榮民總醫院
手術室護理記錄

[redacted]

## 基本資料

- 室別： 開刀房-02室，手術科別： CRS，手術類別： 常規，日期： 2020/04/13
- 進入時間： 0735 ； 麻醉開始時間： 0745 ； Time Out時間： 0940 ； 劃刀時間： 0941 ； 手術結束時間： 1235 送出時間： 1300 ；
- 麻醉方式： GE
  診斷： Malignant tumor of DISTAL T -colon, TRY LAP
  手術名稱： Laparoscopic Anterior resection and anastomosis - malignant
  手術局部用藥： Nil.
  術前預防性抗生素：
  - 抗生素名稱一： Veterin 1GM IVD由麻醉護理師執行
  - 時間： 0910
  - 上次簽章: 黃素惠

  ，追加預防性抗生素：
- 手術醫師： 蔣鋒帆，林昌霖，洪浚嚴
- 麻醉醫師： VS 李淑娟
- 
  - 刷手護士A ： 詹佳穎，時間： 0735，接替刷手一： 黃素惠，時間： 1230
  - 流動護士A ： 黃素惠，時間： 0735，接替流動一： 許育綾，時間： 1200，接替流動二： 詹佳穎，時間： 1300

---

## 皮膚狀況

A-V Shunt： 無
術前： 完整
記錄者： 上次簽章: 黃素惠
術後： 完整
記錄者： 詹佳穎

---

## 手術準備

手術臥式 單一手術臥式

- 
  - 手術臥式： 仰臥，截石臥式
  - 使用附件： 矽膠枕頭
  - 皮膚準備： 10%Aq Betadine，Hibiscrub，其它： 2% Chlorhexidine
  - 電燒： 使用電燒: 電極片位置： 左大腿，記錄者： 上次簽章: 黃素惠

止血帶T： 無

---

## 植入物

- 植入物名稱/號碼/數量/部位： Endo GUA 60/3.5mm x3/T-S colon

, 記錄者： 上次簽章: 黃素惠

---

## 標本送檢

- 細菌培養 無
- 細胞學 無
- 冰凍切片 無
- 病理標本
  組織名稱一：T-S colon
- 截肢 無

標本交付 標本： 1 袋，標本： 0 盒，標本： 0 瓶，細胞學： 0 管，Culture Tube： 0 管，被交付醫師： R 洪浚嚴，

- 記錄者： 上次簽章: 許育綾
- 時間： 1235

## 傷口術後敷料

傷口敷料：　　　　　　　　　　傷口敷料： 無

- 請於圖上標示部位：

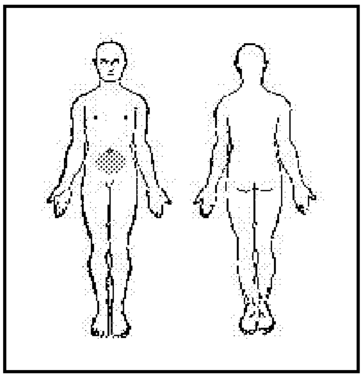

- 傷口敷料： MENPORE

## 導管

| 導管 | 天數 | 導管資訊 | 插入日期 | 插入時間 | 更換日期 | 拔除日期 | 拔除時間 | 拔除原因 | 預計拔除/更換 | 預計拔除/更換日期 |
|---|---|---|---|---|---|---|---|---|---|---|
| Penrose Drain(1) | 1 | 號碼:12 mm.; 部位:脾窩; | 2020/04/13 | 1150 | | | | | | |
| Foley(1) | 1 | 號碼:16 Fr.; 部位:膀胱; | 2020/04/13 | 0900 | | | | | | |
| A-line (1) | 1 | 號碼:2.G. ; 部位:右橈動脈; | 2020/04/13 | 0835 | | | | | | |
| Sump NG Tube(1) | 1 | 號碼:16 Fr.; 深度:55CM; 部位:胃; | 2020/04/13 | 0930 | | | | | | |

## 用物計數

- 手術中出血量： MINIMAL c.c.
- 手術中尿量： 500 c.c.
- 
    - 其它： 無

用物計數： 多切口同時縫合

- 1.器械： 正確，2.縫針： 正確，3.刀片： 正確，4.紗布： 正確，5.腹部墊： 未使用，6.紗球： 未使用，7.PEANUTS ： 未使用，8.紗條： 未使用，9.綵帶： 未使用，10.針頭： 未使用，11.Vessel loop： 未使用，12.Boots： 未使用，13.Jelco： 未使用，14.棉條： 未使用，
    - 第一次：
        - 刷手護士 ： 詹佳穎
        - 流動護士： 上次簽章: 許育綾
        - 時間： 1205

  ,
    - 第二次：
        - 刷手護士 ： 詹佳穎
        - 流動護士： 上次簽章: 許育綾
        - 時間： 1215

  ,
    - 第三次：
        - 刷手護士 ： 詹佳穎
        - 流動護士： 上次簽章: 許育綾
        - 時間： 1230

## 備註

備註： 使用腹腔鏡NO.5

- 記錄者： 上次簽章: 黃素惠
- 時間： 1130

填寫護理人員 詹佳穎

正式摘要

| | |
|---|---|
| | W52 / W52 |

doctorRank:B
